# Both Loved and Feared: Third Party Punishers Are Viewed as Formidable and Likeable, but These Reputational Benefits May Only Be Open to Dominant Individuals

**DOI:** 10.1371/journal.pone.0110045

**Published:** 2014-10-27

**Authors:** David S. Gordon, Joah R. Madden, Stephen E. G. Lea

**Affiliations:** School of Psychology, University of Exeter, Exeter, Devon, United Kingdom; University of Jyväskylä, Finland

## Abstract

Third party punishment can be evolutionarily stable if there is heterogeneity in the cost of punishment or if punishers receive a reputational benefit from their actions. A dominant position might allow some individuals to punish at a lower cost than others and by doing so access these reputational benefits. Three vignette-based studies measured participants' judgements of a third party punisher in comparison to those exhibiting other aggressive/dominant behaviours (Study 1), when there was variation in the success of punishment (Study 2), and variation in the status of the punisher and the type of punishment used (Study 3). Third party punishers were judged to be more likeable than (but equally dominant as) those who engaged in other types of dominant behaviour (Study 1), were judged to be equally likeable and dominant whether their intervention succeeded or failed (Study 2), and participants believed that only a dominant punisher could intervene successfully (regardless of whether punishment was violent or non-violent) and that subordinate punishers would face a higher risk of retaliation (Study 3). The results suggest that dominance can dramatically reduce the cost of punishment, and that while individuals can gain a great deal of reputational benefit from engaging in third party punishment, these benefits are only open to dominant individuals. Taking the status of punishers into account may therefore help explain the evolution of third party punishment.

## Introduction

Third party punishment has been consistently shown to be one of the main factors that ensures cooperation within groups of individuals [1]. Third party punishment is effective at promoting cooperation even if it is delayed [2] or given in a verbal form only [3], and the mere presence of a third party significantly increases both fair behaviour and, conversely, the unwillingness to accept unfair behaviour [4]. Third party punishment has also been claimed to be a universal human behaviour [5]. Nevertheless, there is continuing debate as to how third party punishment could initially evolve, because it imposes costs on an individual while the benefits are shared amongst the group as a whole [6]. In this paper, we consider two, not mutually exclusive, solutions to this problem: that individual's gain indirect benefits from punishment, and that certain individuals can punish at reduced cost.

### 1.1 Reputation and third party punishment

The problem of the cost individuals pay for punishment can potentially be overcome if there was some way for punishers to recover the costs of punishment through indirect benefits from their actions. Specifically, through earning a positive reputation [7], [8]. Third party punishers are seen as trustworthy, group focused and ‘nice’ [9], [10] and these positive social attitudes translate into actual rewards. It has been suggested that engaging in third party punishment can act as a costly signal of an individual's altruistic nature [10] and, indeed, those who punish are often also highly cooperative [11]. Interestingly, the positive traits associated with punishment are also those we demand in leaders [12], and with this in mind the results of Gürerk et al. [13], that individuals prefer an environment where punishment is possible, could be reinterpreted to suggest individuals prefer to be in an environment where *someone* will punish social defection. A reputation as a punisher might therefore allow the punisher to recruit social allies or cooperative partners more easily because, as well as signalling their own altruistic and cooperative tendencies, it may suggest they will intervene to ensure any individual in their vicinity is treated fairly.

Alternatively, engaging in punishment might be less about signalling pro-social personal characteristics and more about signalling dominance and personal formidability. Third party punishment, if not an aggressive act *per se*, can certainly be considered a confrontational one as at some point it must involve an individual attempting to inflict a cost upon a defector or aggressor, and most confrontational actions are instigated by dominant individuals [14]–[16]. Humans can easily identify the victor in a confrontation [17] and recognising one's place in a dominance hierarchy is a vital part of the social cognition of any group-living animal [18]. Accordingly, engaging in punishment would certainly suggest a dominant position. Indeed, the examples of punishment that occur in the non-human literature are carried out by dominant individuals only [19]–[21], with the apparent purpose of maintaining their social rank.

Benard [22] showed that aggressive behaviour does act as a deterrence against confrontational behaviour, and it has been suggested that ‘cooperative’ behaviour could be a result of coercion by a more powerful receiving party [23]. In fact, Marlowe et al [24] suggested that one reason for the lack of third party punishment in small scale human societies is that, due to eavesdropping on dyadic interactions, a ‘don't mess with me’ reputation can be easily established without an individual involving themselves in the conflicts of others. Thus third party punishment could be another form of aggression used as a signal of social position and to demonstrate personal formidability, i.e. ‘don't mess with the enforcer’ [9], with any rewards from the behaviour [10] being due to fear. Nevertheless, the reputation gained from an act of third party punishment need not only be either as trustworthy person or as a formidable person; it could be both. For example, research on welfare trade-off ratios, the process by which we make resource allocation decisions [16], splits the factors in this process into two broad categories: the potential benefit the recipient provides to us, and their ability to inflict costs upon us. Thus an act of punishment could provide duel social gains to a punisher because, on the one hand, they are seen as beneficial to be around as their actions indicate they are trustworthy and are willing to defend group norms and eliminate free-riders, and on the other hand they have signalled their individual formidability or willingness to use force and thus should be treated fairly or even with deference.

However even if there are benefits are available in the long-term from engaging in punishment, the immediate costs of punishment (see below) still present a barrier of entry for this behaviour [25], [26]. While reputational gain may offset the cost of punishment indirectly, this will only occur if the punisher survives the attempt at punishment itself. But if there is heterogeneity in the cost of punishment, the behaviour can emerge even without reputation being a factor [27], [28], and then be further stabilised by the subsequent reputational gain. We believe such heterogeneity can be represented by position in a dominance hierarchy, and that a dominant position not only lowers the immediate cost of punishment, but in doing so allows dominant individuals-only to access the indirect/reputational benefits of third party punishment.

Dominance itself can be difficult to define [29], here we use the term loosely to cover a range of concepts such as formidability, status, prestige and power; i.e. simply as a label for an individual who has a strong position is a social hierarchy, or who is recognised as having *“priority of access to resources”* [18]. While there are likely to be nuanced differences between types of status, we believe that the benefits of a ‘strong social position’ as described in the current article would be comparable whether this position was achieved through, for example, aggression or prestige [30].

### 1.2 Dominance and the cost of punishment

Why would differences in dominance translate into heterogeneity in the cost of punishment? Firstly, dominant individuals have access to a greater amount of resources [18]. For example, their position gives them greater opportunities for reciprocity and cooperation [31] and their prominence means that others are both willing to tolerate asymmetries in reciprocity and to provide aid in conflicts in order to maintain a close relationship with the dominant individual [32], [33]. Dominant individuals also demand that their needs are met above others [16], can behave coercively in dyadic relationships to ensure this [34], and are less likely to face punishment for behaving unfairly [4], [35]. Because of their higher total resources, a given act of punishment costs a dominant individual a smaller fraction of their resources,

Secondly, dominance may reduce the cost of punishment by making it more effective, i.e. by making the cost it inflicts on the punished individuals higher. Effectiveness of punishment is important to its evolutionary stability [27], and only effective punishment has been shown to deter free-riding [25]. However, while this finding is consistent across the experimental third party punishment literature, so far little has been said as to how this would manifest outside of the laboratory, i.e. what would allow an individual to punish effectively? Dominant individuals, we argue, can punish more effectively, insomuch as they can inflict a greater cost on the target either physically [16] or by using their social position to limit access to resources or information [36].

Furthermore, perhaps the most important cost to third party punishment is retaliation from the target [37]. Where retaliation to punishment is possible, third party punishment is reduced to the point that it no longer sustains cooperation or is evolutionarily stable [37], [38] and, in everyday life, the threat of retaliation is a prime factor in preventing otherwise cost-free punishment behaviour such as reporting criminal activity [39]. Dominant individuals are, self-evidently, successful in dyadic conflicts and, as previously stated, in essence third party punishment is a dyadic interaction between the third party and the defector/norm-violator. Therefore dominant individuals may be able to engage in third party punishment without the risk of reprisals as the target will simply acquiesce to their demands. Indeed, when third party punishment does occur outside the laboratory it is carried out by formidable individuals [40] or by those with the support of allies [41] - circumstances where the threat of retaliation would be reduced. In fact retaliation could be a conventional cost to punishment that may make it a costly signal of either formidability or a pro-social attitude, as even if the production cost of punishment is cheap; for example punishment by condemnation [3], by gossip [42], or by ostracism [43], the retaliatory cost may be severe for anyone in a subordinate position.

Finally, as dominant individuals can punish more effectively and face less risk from retaliation, it may be possible for them to lower the cost of third party punishment still further, potentially to effectively zero, by establishing a credible threat of punishment [44]. Once a reputation for third party punishment has been established, an individual may never, or at least rarely, need to actually engage in punishment for the foreseeable future.

### 1.3 The current studies

From the literature introduced in 1.1 and 1.2 it can be said that while there might be reputational benefits, both for a pro-social nature and from signalling dominance, from engaging in third party punishment, perhaps these benefits can only be accessed by individuals who can overcome the initial costs of punishment; dominant individuals. To investigate this, using a series of vignettes the current article measured social judgments made by uninvolved observers about individuals who engage in third party punishment and what factors affected these judgements. Specifically, we investigated whether dominance judgements are in fact made about third party punishers, and whether judgements of dominance and likability (i.e. ‘pro-social’ attitude about a punisher) were unique to third party punishment or were similar across other antagonistic encounters (Study 1); whether observer judgments of a third party are sensitive to the potential immediate costs of the behaviour (Study 2); and whether a dominant position is recognised as lowering the cost, and raising the likely success, of third party punishment (Study 3).

The majority of work in this area has been conducted using economic games. However the vignette method, as well as being used consistently in social psychology, has also been used to study other phenomena related to human evolution, for example altruism [45], mate choice [46] and formidability [47]. For research on norm violations and a general discussion on the use of vignettes, see [48]

### 1.4 Ethics statement

These studies were conducted in accordance with Ethical Guidelines and with full ethical approval of the University of Exeter Departmental Ethics committee. Participants gave informed written consent before taking part in the studies being presented and were fully debriefed once the studies had been completed. The data set for these studies can be accessed from http://hdl.handle.net/10871/15639.

## Study 1

This exploratory study investigated whether observers judge individuals who engage in third party punishment differently to those who engage in other types of aggressive behaviour, i.e., whether any judgements of dominance or reputational benefits are related to the act itself or, more generally, to an effect of aggression/winning a physical contest.

### 2.1 Method

#### 2.1.1 Participants

414 (132 male) undergraduate students from the University of Exeter, UK, successfully completed the survey. Participants were recruited via email using an existing ‘paid participant’ list. As an incentive to take part, any participant who completed the survey was entered into a prize-draw for a number of store vouchers worth £10 (about US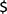;13). The mean age of participants was 22 years. 25 participants failed manipulation check questions and their data was excluded from all analyses

#### 2.1.2 Materials and procedure

The survey was administered online. Participants followed an email link which randomly assigned participants to a condition which presented them with a survey consisting of two sections. They were first presented with the experimental vignette, described and presented as a ‘news website-style article’. It was not made explicit whether this article was real or fake. To keep with the ‘news site’ aesthetic and the wider aims of the study, the article included a picture of its subject, a male identified only as ‘John Taylor’. This picture was chosen from a set of photos collected for a previous study [49] as the face received neutral ratings in regards to attractiveness and trustworthiness. Once participants had finished reading the article they were presented the second section of the survey which contained a series of questions concerning John.

#### 2.1.3 Experimental Scenario

Participants were presented with one of four possible articles concerning the actions of John. In the Third Party punishment condition, John was described as having successfully intervened to stop the mugging of an old man late at night; in the Second Party punishment condition, John was described as having successfully fought off a mugger late at night; in the Random Fight condition, John was described as having been involved in a bar fight of indeterminate cause; and in the Control condition John was described as having witnessed a flash-mob. In all three experimental conditions the assailant who fought John was described as “a 6ft muscular male”. For the full scenario, see File S1.

#### 2.1.4 Social questions

Participants were asked a series of questions regarding how likable John was. They were asked to rate John on a scale of 1 (strongly disagree) to 7 (strongly agree) as to how trustworthy, group focused and ‘nice’ he was, and whether they would work and socialise with him. These questions were adapted from Barclay [9] and in the current study the five items had a high reliability index (α = 0.91). Therefore they were collapsed into a single ‘likability’ variable for all future analyses.

Male Participants then answered a further set of questions concerning how dominant they perceived the third party to be by rating him, on a scale of 1–7 (1 = strongly disagree, 7 = strongly agree), on how threatening, intimidating, dominant, antagonistic or aggressive he was. These questions were adapted from Buss [50] and were also found to have a high reliability index (α = 0.86) and were therefore collapsed into a single ‘dominance’ variable for all future analyses.

As part of the wider aims of the study, female participants (n = 282) were asked questions concerning their willingness to be romantically involved with John (these data are not reported here). In order to keep the questionnaires to a similar length for both sexes, females were not asked to judge John for perceived dominance (see below).

### 2.2. Results

#### 2.2.1 Likeability

**Figure 1:**
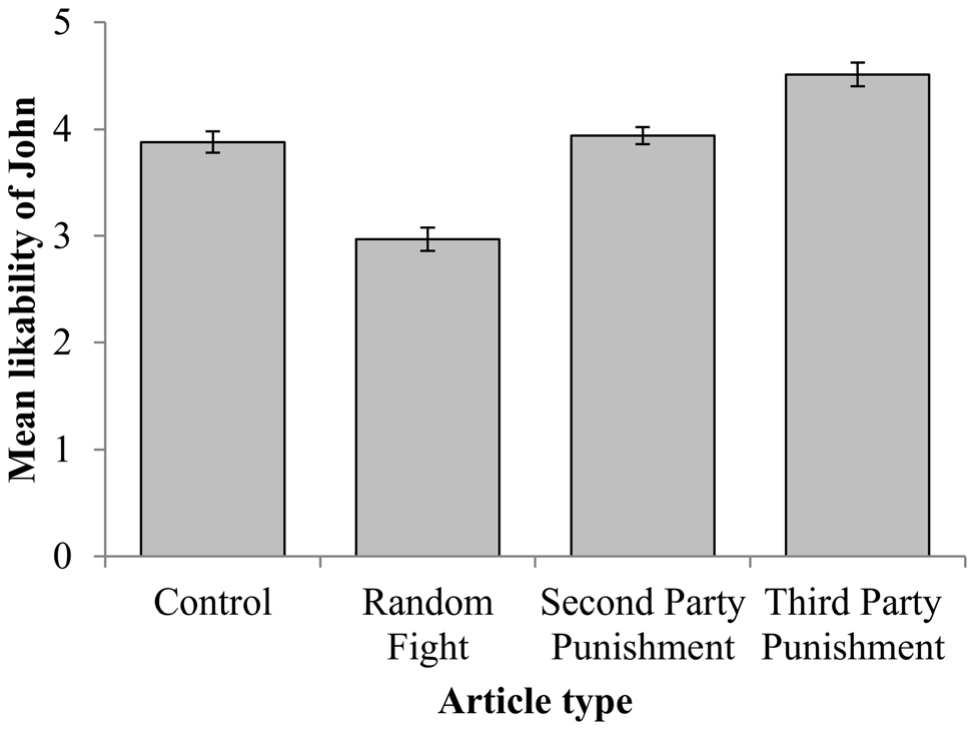
Participant's judgement of John's likeability depending on the type of behaviour he was reported having engaged in. Bars = 1 Standard Error.

As shown in Figure 1, John was seen as more likable when he was depicted as engaging in third party punishment than in the other conditions (*F_3,407_* = 37.46, *p*<0.001; note that three cases were dropped from this analysis as participants had not completed all the ‘likability’ measures). John in the Random Fight condition was the least liked. Bonferroni-corrected pair comparisons found significant differences (all p<0.001) between all pairs of Article-types except between the Control and Second Party conditions (p = 1.0). The sex of the participant did not affect overall judgements or interact with the type of scenario presented to participants.

#### 2.2.2 Social Dominance

John was judged as more dominant in all the experimental conditions compared to the Control condition (Third Party, M = 3.42, SD = 1.10; Second Party, M = 3.34, SD = 1.01, Random Fight, M = 3.55, SD = 1.05, Control, M = 2.57, SD = 0.94; *F_3,128_* = 5.78, *p* = 0.001). Bonferroni-corrected pair comparisons found significant differences between the Control Article and the experimental conditions (Random Fight, p = 0.002; Second Party Punishment, p = 0.008; Third Party Punishment, p = 0.01), but no differences in judgments of dominance between the three experimental conditions (all p = 1.0).

### 2.3. Discussion

These results show that the increase in likability of third party punishers cannot be explained alone by them winning an altercation or by the ‘warm glow’ that may accompany seeing an offender receive retribution [51], [52]. When John fought off his own attacker, he was seen as no more likable than in the control article where John did nothing. This is likely because second party punishment is driven by a desire to protect oneself, or for personal retribution and to save face [53]. Thus defending yourself says little about your qualities save your ability to fight back. That John was seen as most likable when engaging in third party punishment adds further evidence to the suggestion that the behaviour can signal specific additional information about the altruistic and trustworthy nature of a punisher [10]. Indeed, while there can be sex differences in how violence is perceived [54], both males and females made similar judgements about the likability of John.

Judgements about dominance were however dependent solely on the aggression in the encounter rather than on the context, i.e., John was seen as equally dominant whether he intervened as a third party or was involved in a fight with an indeterminate cause. This is unsurprising as engaging in aggressive behaviour is a signal of dominance [15] and perceiving dominance from an interaction can be seen as a reasonably objective process; it is in our interests to make accurate observations of a social hierarchy [18] and the outcome of a confrontation can be easily recognised [17]. It should be remembered that the dominance data came from male participants only, however for the aforementioned reasons, i.e. that it is in all individuals interest to accurately assess dominance, and because it has been shown that both males and females agree on male formidability [55], it is unlikely that dominance judgments would be affected by sex.

By comparing the judgements of a third party punisher to other aggressive acts, this study demonstrated that engaging in third party punishment alone provided the punisher with positive reputational benefits. This study also demonstrated, in males at least, that engaging in third party punishment can make one seem more dominant without the negative social consequences associated with other forms of aggressive behaviour.

## Study 2

Study 1 found that third party punishers are judged to be more likable than individuals who engage in other aggressive behaviours, yet were judged to no less dominant than individuals who engage in other aggressive acts. Study 2 also investigated what information observers are using to judge third party punishers, specifically whether judgements are affected by the success of the intervention and whether the level of threat an aggressor posed would further affect a participant's perceptions of the punisher. Study 2 also investigated whether these factors affected the perceived dominance rank of the aggressor/defector, the victim and the third party relative to one another, i.e., if punishment can signal a dominant position in a group.

### 3.1 Method

#### 3.1.1 Participants and materials

102 psychology undergraduate psychology students from the University of Exeter (85 females) successfully completed the study, with an additional 12 participants either failing the manipulation checks or dropping out of the study before completion. Participants were recruited via email from the 1^st^ year psychology cohort (2011). As an incentive to take part, any participant who completed the survey was entered into a prize draw for a number of online-store vouchers worth £10 (about 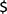
13 US). The mean age of participants was 21. The study employed a between-subjects design with 3 experimental conditions and one control condition; participants followed an email link which randomly presented with one of four experimental vignettes, followed by a series of questions concerning the third party punisher in these vignettes.

#### 3.1.2 Experimental Scenario

Participants were asked to imagine themselves seated alone in a local bar and told that they observed a group of men enter and occupy a table nearby. Participants were then told they observed an altercation between group members in which one member (the ‘aggressor’) forced another (the ‘victim’) to relinquish his seat so the aggressor could sit down. These labels are for clarity only; in the scenario itself the characters were identified by the colour of the shirts they were described as wearing.

In condition 1, the ‘successful’ condition, a third group member (the ‘third party’) successfully intervened and forced the aggressor to give back the seat. In condition 2, the ‘unsuccessful’ condition, the third party intervened but failed to force the aggressor to give back the seat. In condition 3, the ‘increased threat’ condition, participants were told they observed a successful act of punishment, but in this scenario the male characters were unknown to one another and not part of a self-contained group. Thus, because the third party did not have prior social knowledge of the aggressor, and because the latter could have ‘targeted’ the participant/observer, the aggressor was a greater potential threat in this scenario. This increased threat condition matched the successful condition in all other respects. In condition 4, the ‘control/no action’ condition, participants were told they observed the interaction as in Condition 1 & 2, but here the third party became agitated but did not intervene. For the full scenario, see File S1.

#### 3.1.3 Social Questions

Participants were then asked to make a series of social judgements about the third party in the scenario. Firstly, participants were asked to rank the three characters in the story in terms of dominance (1 being most dominant and 3 being least dominant). All participants were then asked the five likability questions (α = 0.88 for this study) and the five social dominance questions (α = 0.85 for this study) as described in Study 1. As in Study 1 these items were collapsed into single ‘likability’ and ‘dominance’ variables for all future analyses.

### 3.2 Results

The study tested two distinct hypotheses: that there would be a relationship between how participants responded to a third party depending on their level of intervention (successful vs. unsuccessful vs. control; N = 82), and that there would be a difference in participant responses between the level of threat posed by the aggressor (successful vs. increased threat; N = 59). Data relating to these hypotheses were analysed separately.

#### 3.2.1 Third Party behaviour and relative dominance rank

**Figure 2:**
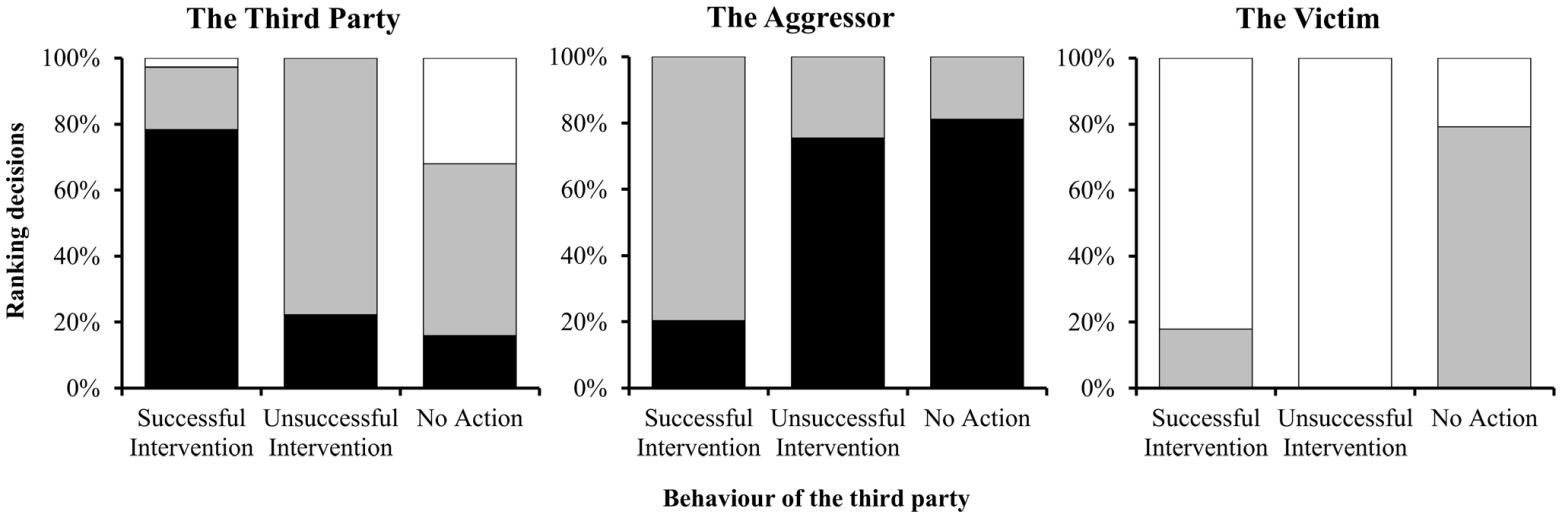
Proportion of participants who, across conditions, ranked the third party, the aggressor and the victim as the most dominant character (black bars), gave the character the middle rank (grey bars) or ranked them as the least dominant character (white bars).

Participants ranked the third party to be most dominant when he successfully intervened, with fewer ranking him as most dominant when the intervention failed, and the fewest when he did not intervene. The victim was nearly always ranked as least dominant (Figure 2). To investigate the relative difference between the characters, we considered which character was ranked as the most dominant by participants. The success of punishment affected whether the third party was seen as the most dominant individuals (*χ^2^_2_* = 28.75, *p*<0.001): In the successful condition, the third party was more likely to be ranked as most dominant (78%), compared to the third party who unsuccessfully (22%) or failed to (19%) intervene.

#### 3.2.2 Third Party behaviour and judgements of dominance

**Figure 3:**
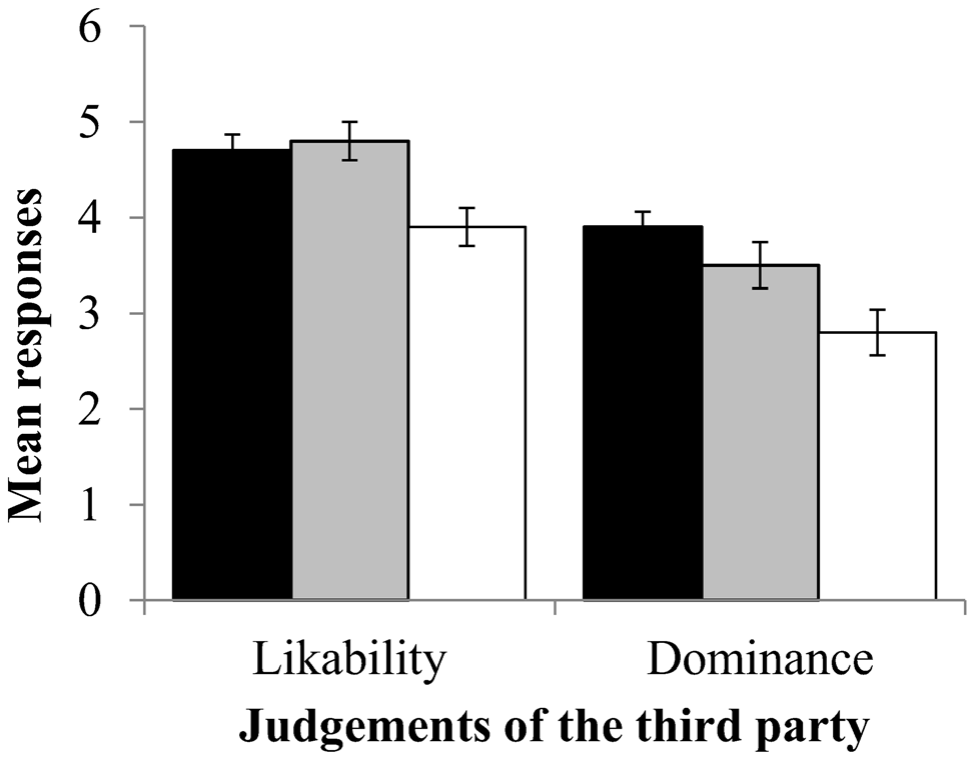
Judgement of likeability and dominance of the third party depending on whether the third party successfully intervened (black), unsuccessfully intervened (grey) or failed to intervene (white). Bars = 1 Standard Error.

Figure 3 shows that the third party was judged to be more dominant when he attempted to intervene, regardless of whether or not he was successful, than when he did not interviene (*F_2,79_* = 7.16, *p*<0.001, Contrast analysis: successful vs. unsuccessful, *F*_1,80_ = 1.65, *p* = 0.20; successful vs. no action, *F_1,_*_ 80_ = 14.30, *p*<0.001; unsuccessful vs. no action, *F_1,_*_ 80_ = 4.06, *p* = 0.047).

#### 3.2.3 Third Party behaviour and judgements of likability

Figure 3 also shows that the third party was judged to be more likeable when he attempted to intervene, regardless of whether or not he was successful, than when he did not intervene (*F_2,80_* = 4.72, *p* = 0.009; Contrast analysis: Successful vs. Unsuccessful, *F_1,81_* = 0.15, *p* = 0.70; Successful vs. No Action, *F_1,81_* = 7.27, *p* = 0.009; Unsuccessful vs. No Action, *F_1,81_* = 6.40, *p* = 0.01).

#### 3.2.4 The threat posed by the attacker and judgements of the third party

The third party was judged to be more dominant when the threat posed by the aggressor was increased (M = 4.56, SD = 1.11) compared to the successful condition (M = 3.95, SD = 1.02; *F*_1,58_ = 4.56, *p* = 0.037). However, the level of threat did not affect how likable the third party was judged to be (increased threat, M = 4.80, SD = 1.21; successful, M = 4.70, SD = 1.09; *F_1,58_* = 0.11, *p* = 0.75).

### 3.3 Discussion

Here, the results concerning the judgements of dominance are unequivocal; successful intervention by the third party led participants to perceive him as most dominant, and unsuccessful intervention led to the aggressor being perceived as most dominant. While this result is no surprise, to the authors' knowledge it is the first study to demonstrate that engaging in third party punishment directly affects the perceptions of an uninvolved observer with respect to the punisher in this manner.

What is surprising is that when asked to make social judgements about the third party, participants judged him to be more likeable and dominant when he intervened, regardless of the success of the intervention. While it has been demonstrated that there are reputational gains from engaging in third party punishment [9], [10], in these studies, as was the case in the vignette for Study 1, by design punishment was always successful. That perceived likeability and dominance remained even when the interaction was unsuccessful suggests that such ratings are not due to a halo effect of seeing an antisocial individual punished [51], [52] or due to the punisher being the recipient of indirect or strong reciprocity for carrying out a public function.

The results do however add further evidence to the suggestion that third party punishment can be seen as a costly signal of dominance. Due to the threat of retaliation [38], [56], the mere act of punishment should provide an honest signal, as retaliatory costs will likely be present whether the intervention was successful or not (see Study 3). This is further highlighted by the higher dominance rating given to the punisher in the Increased Threat condition; the lack of any social information or social support from fellow group members made the risks even higher and thus the signal more reliable.

However, there was no corresponding increase in likability in the higher threat condition. Nelissen [10] suggested that increased signal reliability should increase the positive attitude to the punisher and the lack of an effect here may suggest there is an upper limit to the positive attitude engaging in punishment generates: the motivations of punishers might be questionable [9], [57] and, in this study at least, punishment was aggressive and aggressive individuals are generally disliked [58].

## Study 3

Both Study 1 and 2 found that third party punishment increased an individual's likability and perceived dominance and these are both benefits that could allow a punisher to recuperate the cost of punishment. Punishment can also be evolutionarily stable if the cost of punishment is low [27], and this can be achieved if a threat of punishment is credible [59] or through less aggressive punishment such as ostracism [43]. Study 3 therefore addressed whether the status of a third party punisher affected the perception of their ability to make the threat of punishment credible, whether it affected the risk of retaliation they faced, and whether the type of intervention affected how punishers are judged. Study 3 also addressed how these factors affected any reputational gains generated from an act of punishment.

Also in response to the results of Study 2, the scenario was altered (see below) to lower the ‘risk’ to participants from the aggressor: participants were described as being within the group and the targets for aggression were out-group members. These changes also allowed the information regarding status to be integrated into the vignette more subtly.

### 4.1 Method

#### 4.1.1 Participants & Materials

108 psychology undergraduate students from the University of Exeter (86 females) completed the study in 2013. Participants were not offered any incentive for taking part. The survey was administered in paper-form by a single researcher who approached potential participants in and around the Psychology building. Those who agreed to take part were presented with a paper questionnaire containing one of four experimental vignettes and a series of questions concerning the Third Party punisher in the scenarios. Prior to the questionnaire being given to the researcher, the order of administration was randomised using the random-number generator feature of Excel.

#### 4.1.2 Experimental Scenarios

Participants were asked to imagine themselves as part of a local sports team, who, following an evening practice session, had retired to a local bar. The team had occupied a table but there were not enough seats for everyone so some members, including the participant, had to stand. Nearby, two strangers were sitting at another table and after a few minutes one of them headed to the bar to order drinks. Seeing this, one of the standing members of the team went over to the table and proceeded to take the now vacant chair, dismissing the objections of the still seated stranger. Upon their return with the chair, another member of the team confronted this person about their actions.

The study manipulated the status of the confronting team member – the third party – and how they carried out their confrontation (Punishment Type). They were described as either “popular and the most skilled player” (dominant) or “unpopular and the least skilled player” (subordinate), and they either threatened to hit the other team member (physical punishment) or threatened to prevent them playing in all future matches (social punishment), giving the study a 2×2 between-subjects design.

‘Dominance’ in a social group, especially human groups, does not depend solely on formidability [60], and we have used it to describe a person recognised as having a strong social position, or as “having priority access to resources” [18]. Therefore for Study 3, we operationalised dominance to mean a skilled/prestigious position. This allowed us to manipulate the type of punishment, as a prestigious individual can potentially punish effectively by using social, as opposed to physical, power. For the full scenario, see File S1.

#### 4.1.3 Social perception questions

Following the scenario, participants were first asked a series of questions designed to investigate how credible the threats from the third party were. Participants were asked to indicate ‘what happened next’ from one of two choices; either the punishment was successful with the team member returning the chair, or unsuccessful and the team member kept the chair. They were also asked to indicate on a scale of 1–7 (1 = not surprised, 7 = very surprised), how surprised they were that the specific individual in the scenario intervened and, on a scale of 1–7 (1 = very unlikely, 7 = very likely), whether they believed the reprimanded individual would retaliate against the punisher. All participants were then asked the five likability questions (α = 0.82 for this study) and the five social dominance questions (α = 0.85 for this study) as detailed in Study 1.

### 4.2 Results

#### 4.2.1 Credible threat of punishment

**Figure 4:**
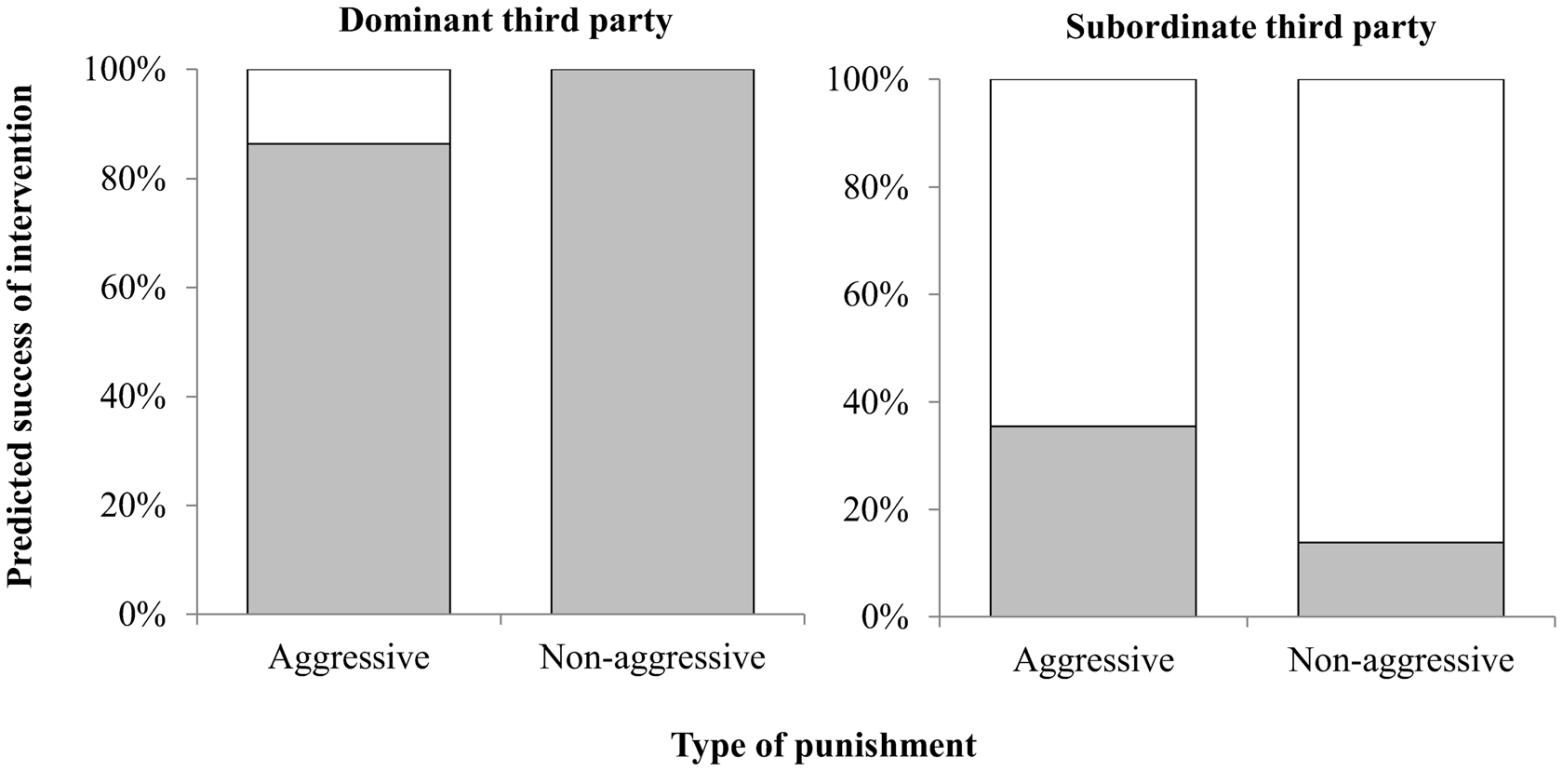
Proportion of participants who believed that a) dominant or b) subordinate punisher would be successful (grey) or unsuccessful (white) in their attempt at punishment.

Participants were first asked whether they believed the aggressor would ignore or give in to the Third Party's demands. Participants believed that the intervention by the dominant punisher would be more successful (Wald χ^2^_1_ = 147.53, p<0.001), with the dominant third party predicted to be successful by 94% of participants, whereas the subordinate was predicted to be successful by 22%. Participants did not believe that the type of punishment alone would alter the outcome (Wald χ^2^_1_ = 0.51, p = 0.48). Figure 4 shows that while participants believed the dominant punisher would be successful regardless of punishment type, the subordinate punisher was thought to be successful only when being physically aggressive (Wald χ^2^_2_ = 9.80, *p* = 0.002).

**Figure 5:**
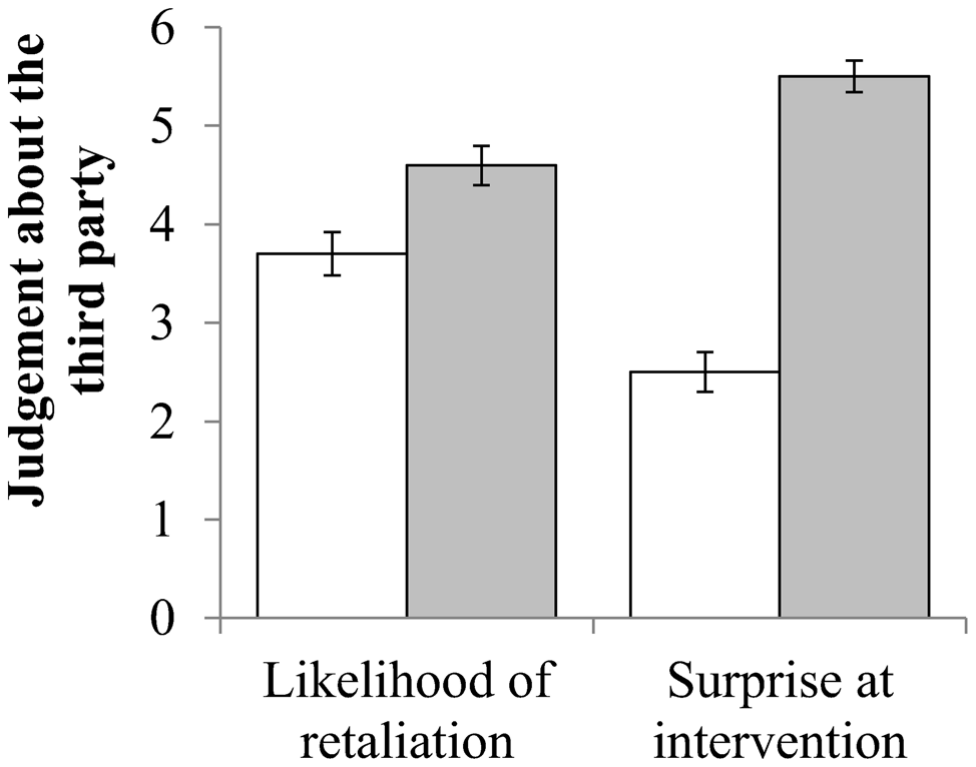
Participants' reaction to the intervention for a dominant (white) or subordinate (grey) Third Party. Bars = 1 Standard Error.

As shown in Figure 5, participants were far more surprised when a subordinate third party attempted punishment compared to the dominant third party (*F_1,104_* = 128.16, p<0.001) and believed retaliation from this intervention was more likely to follow (*F_1,104_* = 6.70, p = 0.011). Neither variable was affected by the type of punishment, or by an interaction between dominance and punishment.

#### 4.2.2 Dominance and Likability

**Figure 6:**
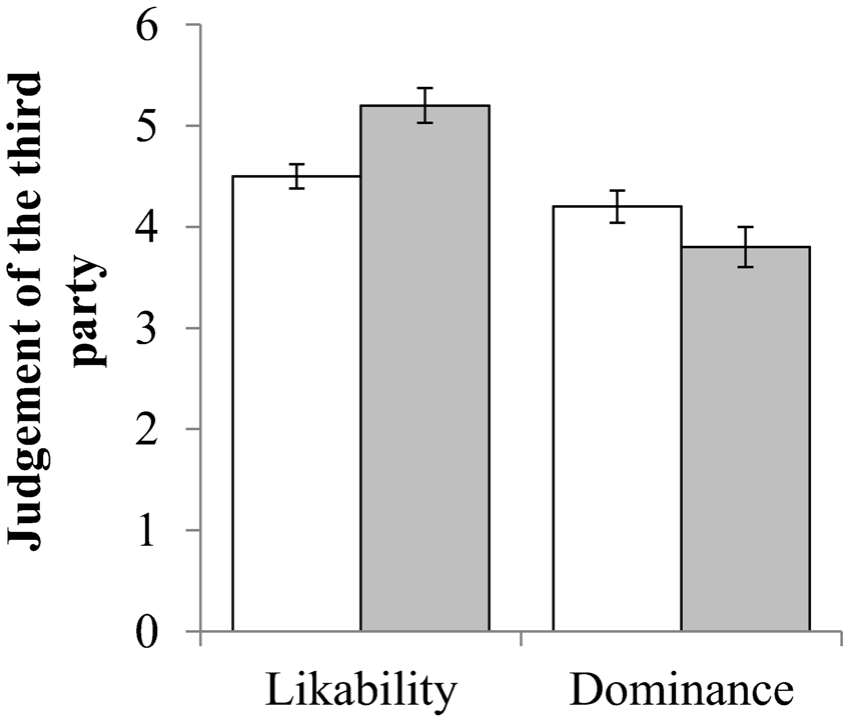
Participants' perception a third party's likability and dominance when they engaged in aggressive (white) or non-aggressive (grey) punishment. Bars = 1 Standard Error.

The dominant third party was, as may be expected, judged to be more dominant (dominant, *M* = 5.5, *SD* = 1.1; subordinate, *M* = 3.6, *SD* = 1.2; *F_1,104_* = 111.76, *p* = 0.001) but there was no effect of dominance on how likable they were judged to be (*F_1,104_* = 0.48, *p* = 0.49). As shown in Figure 6, when the third party engaged in aggressive punishment they were seen as less likable (*F_1,104_* = 6.84, *p* = 0.01): however, being more aggressive did not lead the punisher to be judged as more dominant (*F_1,104_* = 2.07, *p* = 0.10). No interaction was found between either Status and Punishment for likability (*F_1,104_* = 0.83, *p* = 0.77) or social dominance (*F_1,104_* = 0.43, *p* = 0.51).

#### 4.2.3 Success, likeability and retaliation

Given the results regarding the insensitivity of participants to the success of punishment found in Study 2, a post-hoc analysis was carried out to see if there was any relationship between predicted success and likability; none was found (*U* = 1308.5, N_1_ = 59, N_2_ = 48, *p* = 0.5). However, there was a relationship between predicted success and retaliation, with participants believing unsuccessful punishers to be at greater risk from retaliation (M = 4.79, SD = 1.56) than successful punishers (M = 3.73, SD = 1.50; *U* = 856.5, N_1_ = 59, N_2_ = 48, *p*<0.001).

### 4.3 Discussion

These results clearly suggest that dominance can drastically lower the cost of punishment. Firstly, particpants believed that only dominant third parties would be successful in their intervention. Thus, for dominant individuals, the realised costs of third party punishment can be effectively reduced or even removed completely by replacing physical action with a credible threat of punishment. Importantly, the credible threat imposed by the dominant third party was not based on the type of punishment employed; they were seen as equally likely to be successful whether the threat was aggressive (threat of physical violence) or non-aggressive (ostracism from the group). In fact ostracism has previously been shown to facilitate group cooperation without coordinated punishment and at no cost to the punisher [43]. Such a threat therefore can be seen as highly credible, but only if it comes from a dominant individual

Equally, this study found that dominant individuals were judged to be at less risk of retaliation than subordinates. Study 2 established that individuals who attempt third party punishment are seen as more dominant, yet participants in Study 3 were both surprised at the intervention by the subordinate individual and believed they would be at greater risk from retaliation. Even dominant individuals were judged to be at some risk and it may be the case that at least some risk of retaliation is required for any punishment to be a costly signal: while their threats may be credible, a dominant individual would need to prove on occasion they can actually enforce such threats. Indeed, while potentially third party punishment may be important in signalling one's dominant position, participants felt that a subordinate individual attempting to assert themselves in this way would be unsuccessful. In both human and non-human animals false-signalling is often responded to severely [61]–[63] and in the current study participants believed that attempted punishment by a subordinate would lead to a greater risk of retaliation.

The study also suggested that the social benefits generated by engaging in punishment are significantly affected by dominance, specifically the ability to successfully use non-violent punishment. Participants disliked third parties who threatened physical violence in general and only the dominant punisher was perceived as being successful when non-violent punishment was threatened. Dominant individuals can therefore punish in a more socially acceptable way and as a result make greater reputational gains that subordinates. Nevertheless, the dominant punisher in Study 3 was only able to punish so non-violently due to their authority in the groups and this leverage may not always exist in ‘real life’. However, while less liked, the violent stance by the dominant punisher was also predicted to be successful. In comparison to the results of Study 1, where third party punishers were more well-liked in comparison to other violent behaviour, Study 3 suggests that while any third party punishment is responded to positively by observers, there is a preference for less violent intervention.

Study 2 and 3 found that success was no predictor of ‘likability’, but while this does suggest that potentially both dominant and subordinate individuals could gain a reputational benefit from attempting punishment, participants also believed that failure in punishment would invite retaliation; for subordinates, the retaliatory cost of failure would likely outweigh any benefits from the attempt. Again, participants were very surprised at the intervention by a subordinate punisher, so while the vignette ‘forced’ a subordinate to punish, it is debatable whether in a real-life situation a low status or subordinate individual would actually engage in third party punishment.

## General Discussion

The studies above investigated whether variation in dominance within a group might allow third party punishment to be evolutionary stable because a dominant position allows individuals to access the reputational benefits from punish at a lower cost than others. Study 1 demonstrated simply that, when compared to other violent confrontations, third party punishment makes an individual seem both formidable and likable, i.e., that it yields reputational benefits for the punisher. Study 2 demonstrated that it was the attempt at punishment and not its success that led to these reputational gains. Finally, Study 3 demonstrated that dominant individuals could punish ‘cost-free’: not only was any target of punishment perceived as (very) likely to back down before physical action could occur, but dominant individuals were also seen as having a reduced risk of costs from retaliation and could punish in a more socially acceptable way. Taken together, these studies suggest that third party punishment is seen as a dominant act, that any attempt at third party punishment yields reputational gain, but, perhaps more importantly, the reputational benefits are only open to dominant individuals. Only dominant individuals can lower the production costs via the effective use of non-violent and cost-free threats of punishment [27], [43] and only dominant individuals can punish with a reduced risk of retaliation. In fact, the costs of retaliation especially may stretch beyond the initial act of punishment. Humans are adept at reputation scoring [64], and if punishers are ‘scored’ in a similar fashion as altruistic individuals [65], then a reputation for enforcing fairness, while potentially beneficial for attracting some cooperative partners, could act as an reputational badge that may invite aggression from others [61], akin to the sheriff in a Western or the eponymous protagonist of a super-hero film; i.e., the person who needs to be ‘taken out’ to allow the exploitation of others. In this instance a reputation for enforcing fairness might work against a punisher, or at least one who could not resist such future actions.

That only more dominant individuals are able to access the reputational benefits of third party punishment moves beyond the idea that punishment merely signals *fairness* [10]. Only dominant individuals are capable of giving this signal credibly, meaning that the reputational benefits from punishment are inextricably linked to dominance. Specifically, while Study 2 did find that reputational benefits are ‘open to all’, Study 3 suggested that intervention by a subordinate individual was both surprising and potentially costly in terms of retaliation. Retaliation may be the key cost to third party punishment [37], [38] and, although we did not test this directly in the current studies, in light of Study 3 it is questionable whether a subordinate individual would ever actually engage in third party punishment. The results of the studies suggest that dominance/status is an important factor in any calculation of the individual economics of punishment. Punishment can be evolutionarily stable if there is heterogeneity in the ability to punish [27], [66] and we suggest that dominance causes sufficient heterogeneity in both the cost and rewards of third party punishment to make this behaviour evolutionarily stable.

More theoretically, Pedersen et al. [67] recently suggested that any account of the evolution of third party punishment in humans must be relatable to behaviour seen in non-human animals. As previously stated, third party punishment can be seen as an antagonistic dyadic interaction between a third party and the defector/aggressor and, across many species, such antagonistic dyadic interaction are both instigated and won by dominant individuals [14], [15]. More directly in line with the assertion of Pederson et al, in non-human animals, dominance determines third party intervention across numerous taxa, for example in fish queues [21], in fallow deer [68], and in Barbary macaques [69]. These interventions seem to be driven by the need to limit or prevent the rise of a social challenger. Third party punishment can therefore be seen as having an origin in recognising and responding to social challenges [70], with only dominant individuals possessing the ability to act upon this recognition. This is important as, firstly, punishment as a tool to maintain social position provides an additional motivation for an individual to engage in the behaviour over and above any benefit from maintaining group cooperation and, secondly, the benefits punishment provides can be seen as independent from group-level cooperation [56].

Although this suggestion is speculative, the demonstration that dominance plays at least a proximate role in mitigating the costs of third party punishment (at least according to the perception of observers), and perhaps that it is partly motivated by status concerns [36], does forge a link to non-human animal behaviour. In much the same way that human reciprocity and cooperation has a base in the more limited cooperative behaviour of other animals [71], third party punishment in humans can be seen as a more sophisticated version of a non-human animal behaviour related to dominance and status contests, rather than one completely distinct to us as a species.

The current studies investigated the perception of punishers rather than punishment behaviour itself. The perceptions of others are no doubt a consideration for punishers [72], as it is from observers that indirect benefits are generated, we cannot be certain from the present results that an individual in a dominant social position would actually engage in more punishment. However, published data suggests that they will. For example we have suggested that dominant individuals can punish more effectively than others, and it a consistent finding that ‘effective’ punishers (those who can inflict higher costs on defectors) will punish more than ‘ineffective’ ones [73].

Future studies might extend our current results by designing experimental mechanism to explicitly simulate the advantages of a dominant position, for example by varying the cost of retaliation to certain individuals or by providing certain individuals with an unequal share of any group product [74]. It should be noted that, while we have referred to ‘dominant’ individuals throughout, we do not mean to suggest that only an ‘alpha male’ type would ever punish, as different attributes will confer dominance in different groups.

In our vignettes no economic costs were inflicted upon the defectors in the scenarios. The costs were physical in Study 1 (aggressor described as being was physically assaulted), while in Studies 2 & 3 there would still be costs via social humiliation due to being publically shamed for, and forced to retract, an ‘unfair’ behaviour [75]. Such ‘non-monetary’ or ‘verbal’ punishment is seen as third party punishment in the economic literature despite the lack of direct economic costs [3], [76].

In any case the imposition of actual costs is often an anticipated downstream effect of the subsequent action taken by the *target* of any punishment, rather than the immediate impact of punishment. As an illustrative example, if an individual came across someone smoking on public transport (illegal in the UK) and demanded they stop, this would still be an act of third party punishment in the classic Fehr (2004) sense (i.e. the desire and subsequent behaviour to uphold a social norm) even if the smoker apologised and snubbed out the cigarette with no further interaction taking place. In such a situation there is only physical punishment if the ‘intervention’ is challenged. Indeed, Levine, Taylor, & Best [77] showed that violence after the intervention by a third party only occurs after a series of escalating behaviours by the parties involved, each of which gives the opportunity for one party to back down.

In fact, the possibility that punishment costs might not be realised is a core argument as to how dominance affects the cost/benefit of third party punishment: essentially, a position of dominance, with its implied ability to inflict effective costs on others, functions as a credible threat. Our smoker above would be well aware of the potential costs (further social embarrassment and/or a physical confrontation) and would thus choose to acquiesce. We believe that people's understanding of this implication was demonstrated by Study 3, as when faced with a challenge from a dominant individual, the transgressor was predicted to back down rather than have cost of punishment realised.

In conclusion, the current studies support the suggestion that dominance played an important role in the evolution of third party punishment. Punishment is seen as a dominant behaviour yet is distinct from other dominant actions in the sense that punishers are well liked, compared to other aggressors. Dominant individuals were seen as being able to punish effectively and at a lower cost than others, therefore dominant individuals can access the signalling or reciprocal benefits generated by punishment at reduced cost. We suggest that taking dominance into account may help answer some of the questions and debates around the evolution of this behaviour, specifically in terms of how some individuals can overcome the costs of punishment. Variation in individual condition can result in effective and efficient norm enforcement [28], thus at the very least, our results demonstrate that the dominance of the actor could be an important factor in overcoming the proximate costs of third party punishment. However we also believe that these results point to human third party punishment behaviour having an evolutionary origin as a dominance-based behaviour, rather than having evolved to specifically promote cooperation and fairness.

## Supporting Information

File S1 Full vignettes for studies 1, 2 and 3.
(DOCX)
