# Supplementary material for: Both Loved and Feared: Third Party Punishers Are Viewed as Formidable and Likeable, but These Reputational Benefits May Only Be Open to Dominant Individuals
Source: PLoS One. 2014 Oct 27;9(10):e110045. doi: 10.1371/journal.pone.0110045 (PMC4210197; doi:10.1371/journal.pone.0110045)
Supplement: File S1 — Full vignettes for studies 1, 2 and 3. (DOCX) [file pone.0110045.s001.docx]

**Study 1: third party punishment vignette**

A have-a-go hero was today praised by the local community for his bravery in foiling the attempted mugging of an elderly gentleman late on Tuesday evening. Harold White, aged 77, was walking home alone after visiting a friend and must have seemed a tempting target that night. Having confronted his intended victim, the mugger forced Mr White into an alley way off the main street and demanded he hand over any valuables he had. Thankfully help was at hand. A local man, John Taylor (pictured), interrupted the assault, causing the assailant to flee the scene. Speaking to the press, Mr Taylor, had this to say about the ordeal

*“I was walking home after a cinema trip and saw two men arguing ahead of me. Suddenly the younger one wrestled the other into the alley nearby. I knew I had to do something”*

*“I demanded the guy leave Harold alone, we exchanged words and he refused. We struggled and I knocked him to the ground. He got up and ran off, and I called the police”*

Police have praised John’s bravery in preventing a crime being committed but reiterated they advise the public against such actions and to instead call the police . They continue that while in this case no weapon was involved, Mr Taylor potentially placed himself in danger by becoming directly involved.

The assailant, described as muscular and around 6^ft^ in height, is now being hunted by police with local hospitals and clinics being asked to report anyone arriving with broken nose or similar injury. Anyone with information that may be relevant to the case should contact their local police station.

**Study 1: second party punishment vignette**

A local man was praised by the local community today for his bravery after fending off an attempted mugging on Tuesday evening. John Taylor (pictured) was walking home alone after a trip to the cinema with friends and must have seemed a tempting target that night. The unidentified assailant confronted Mr Taylor sometime after 11pm. Mr Taylor had this to say about the ordeal

*“I was walking home after seeing a film and suddenly this guy stepped out in front of me and demanded I give him my wallet and phone. I wasn’t going to just hand over my things”*

*“I demanded the guy leave me alone, we exchanged words and he refused. We struggled and I knocked him to the ground. He got up and ran off and I called the police”*

Police have praised Mr Taylor ’s bravery in defending himself but reiterated they advise the public against such actions. They continue that while in this case no weapon was involved, Mr Taylor potentially placed himself in danger by escalating the confrontation with his attacker.

The assailant, described as muscular and around 6^ft^ in height, is now being hunted by police with local hospitals and clinics being asked to report anyone arriving with broken nose or similar injury. Anyone with information that may be relevant to the case should contact their local police station

**Study 1: ‘pub fight’ vignette**

Today the clean-up began after a fight broke out early on Saturday evening. Police were called after two men began brawling outside a local bar, breaking tables and knocking over passers-by as they went. Eye witnesses report hearing raised voices from the two people involved before the fight broke out. As yet there is no information as to what caused the altercation and footage from the bar’s CCTV cameras does not appear to suggest either man was drunk. By the time police arrived the fight had subsided and one of those involved; Mr John Taylor was arrested. He had this to say

*“I don’t really know what happened. It was early in the afternoon and I got into an argument with this random person. Not even sure what it was about now. The whole thing was stupid”*

*“Not sure who started anything physical, we struggled and I knocked him to the ground. He got up and his friends lead him away”*

Police later released John and are seeking witnesses to help identify the other man involved, described as muscular and around 6^ft^ in height. Local hospitals and clinics being asked to report anyone arriving with broken nose or similar injury and anyone with information that may be relevant should contact their local police station.

**Study 1: control / ‘flash mob’ condition**

The flash-mob phenomena arrived here yesterday as the city centre was ground to a halt by a recreation of the famous Radiohead music video to the hit signal “Just”. For those who don’t know, a flash mob is a random collection of individuals who, after signing up to a website, receive instructions guiding them to a specific place at a specific time. Once the signal is given each person carries out a pre arranged action: from a song and dance number to stripping naked, or in this case lying on the floor. The mob dispersed before any comments from the participants could be obtained. But one onlooker, John Taylor, (Pictured) had this to say

*“It was all a little surreal really. One minute the centre is filled with people the next half are lying on the floor”*

*“I could see some people were confused nervous initially, but it’s just a bit of a fun. And I think everyone eventually saw the funny side of it. Certainly made the day more eventful”*

City officials say that while there is nothing illegal in what the mob did, in future they would prefer some notice of future events in case other members of the public are worried by the spectacle and overload the police with calls.

**Study 2: vignette**

You are sitting alone in a local bar when a group of people arrive together and sit in front of you at a nearby table. The bar is quite full and they soon realise there are more of them than chairs with no opportunity to get more. You see one of the standing people, a man in a GREY shirt, go over to one of the seated individuals and, unhumorously, forcefully demand the seated man gives up his seat. After a few seconds the seated man grudgingly gets up and stands away.

*Successful intervention*: another member of the group, a man in a BLUE shirt, notices this taking place and turns to the man in the GREY shirt, angrily berating him for making the seated guy move. After a pause, the man in the GREY shirt gets up and returns to the other standing people and the other man returns to his seat.

*Unsuccessful intervention:* another member of the group, a man in a BLUE shirt, notices this taking place and turns to the man in the GREY shirt, angrily berating him for making the seated guy move. After a pause, the man in the GREY shirt stares at him and laughs before turning away and starting a conversation with the person next to him.

*No action*: another member of the group, a man in a BLUE shirt, notices this taking place and

turns to the male in the GREY shirt. He then however says nothing to the man in the

GREY shirt before resuming his previous conversation.

**Study 3: vignette**

You have been part of a university sports team for around a year. Following a practice session you and a number of other team members have gone to a local bar. You and the team regularly go to the same bar after practice and the staff always seem happy to have you all there.

Once you arrive, the team claims the one remaining free table. However, as the bar is quite full, there are not enough chairs for everyone. You and a number of others therefore have to stand.

Nearby, two strangers are sitting at another table and after a few minutes one of them heads to the bar to order drinks. Seeing this, one of standing members of your team goes over to the table and proceeds to take the now vacant chair. They laugh off the protests of the other stranger, daring them to take it back, and return to your team’s table with the chair.

You see that another member of your team, who is [*popular/unpopular*] in the team and the [*most/least]* skilled member, has also noticed this interaction. Standing up, this person angrily berates the chair-taker, and tells them they “won’t tolerate this behaviour” and will “[*beat the crap out of them/ make sure they never represent the team competitively again*]” if the chair isn’t returned and should they ever do anything like this in the future
